# Evaluating the Differential Response of Transcription Factors in Diploid versus Autotetraploid Rice Leaves Subjected to Diverse Saline–Alkali Stresses

**DOI:** 10.3390/genes14061151

**Published:** 2023-05-25

**Authors:** Ningning Wang, Yingkai Wang, Chenxi Wang, Zitian Leng, Fan Qi, Shiyan Wang, Yiming Zhou, Weilong Meng, Keyan Liu, Chunying Zhang, Jian Ma

**Affiliations:** 1 Faculty of Agronomy, Jilin Agricultural University, Changchun 130117, China; ningningw@jlau.edu.cn (N.W.); 20210043@mails.jlau.edu.cn (Y.W.); 20210044@mails.jlau.edu.cn (C.W.); 20210040@mails.jlau.edu.cn (Z.L.); 20210057@mails.jlau.edu.cn (F.Q.); 20200055@mails.jlau.edu.cn (S.W.); 20200057@mails.jlau.edu.cn (Y.Z.); mengweilong@mails.jlau.edu.cn (W.M.); 20210038@mails.jlau.edu.cn (K.L.); 2 Key Laboratory of Crop Molecular Breeding, Universities of Jilin Province, Changchun 130117, China

**Keywords:** autotetraploid rice, saline–alkaline stress tolerance, transcription factor, hormone

## Abstract

Saline–alkali stress is a significant abiotic stress factor that impacts plant growth, development, and crop yield. Consistent with the notion that genome-wide replication events can enhance plant stress resistance, autotetraploid rice exhibited a higher level of tolerance to saline–alkali stress than its donor counterparts, which is reflected by differential gene expression between autotetraploid and diploid rice in response to salt, alkali, and saline–alkali stress. In this study, we investigated the expression of the transcription factors (TFs) in the leaf tissues of autotetraploid and diploid rice under different types of saline–alkali stress. Transcriptome analysis identified a total of 1040 genes from 55 TF families that were altered in response to these stresses, with a significantly higher number in autotetraploid rice compared to diploid rice. Contrarily, under these stresses, the number of expressed TF genes in autotetraploid rice was greater than that in diploid rice for all three types of stress. In addition to the different numbers, the differentially expressed TF genes were found to be from significantly distinct TF families between autotetraploid and diploid rice genotypes. The GO enrichment analysis unraveled that all the DEGs were distributed with differentially biological functions in rice, in particular those that were enriched in the pathways of phytohormones and salt resistance, signal transduction, and physiological and biochemical metabolism in autotetraploid rice compared to its diploid counterpart. This may provide useful guidance for studying the biological roles of polyploidization in plant resilience in response to saline–alkali stress.

## 1. Introduction

As sessile organisms, plants are unable to relocate in response to unfavorable environmental conditions. Consequently, they have evolved a plethora of strategies to cope with environmental adversity, ensuring their survival and reproduction. These strategies encompass physiological, biochemical, and molecular adaptations to enable plants to withstand or acclimate to various abiotic and biotic stress factors [1]. Saline–alkali stress is a widespread abiotic factor that impacts plant growth, development, and productivity [2]. It arises from high soil salinity and alkalinity, which can result from natural processes or human activities, and affects plants through several interconnected mechanisms [3,4]. Saline stress is predominantly caused by the presence of high levels of soluble salts, mainly NaCl, in the soil. The primary effects of saline stress on plants include osmotic stress, ion toxicity, and nutrient deprivation. Osmotic stress results from high salt concentrations in the soil, reducing water availability for plant roots. Ion toxicity occurs when excessive amounts of salt ions, such as Na^+^ and Cl^−^, accumulate in plant tissues, disrupting cellular processes. Nutrient deprivation, particularly K^+^ and Ca^2+^ deficiencies, arises due to competition between salt ions and essential nutrients. Alkaline stress, on the other hand, is characterized by high soil pH, typically above 8.5, resulting from an excess of basic cations such as Ca^2+^, Mg^2+^, and CO_3_^2−^. The high pH associated with alkaline stress can directly impact enzyme activity, protein stability, and membrane integrity, leading to impaired metabolic processes and reduced plant growth and development [5]. Additionally, alkaline stress can cause nutrient deficiency, particularly in micronutrients such as Fe, Mn, and Zn, as their availability in the soil decreases with increasing pH [6]. Accordingly, based on the salt content and soil pH values used to identify salinization severity, it was classified into a mild level (salt content < 3‰, pH value 7.1–8.5), moderate level (~3–6‰, pH 8.5–9.5), and severe level (>6‰, pH > 9.5), respectively [7]. Although saline and alkaline stresses share some similarities, such as inducing nutrient deficiencies, they primarily affect plant development through different mechanisms. The primary effects of saline stress are related to osmotic and ionic imbalances, while alkaline stress primarily involves pH-induced changes in nutrient availability and toxicity. Understanding the distinct molecular mechanisms underlying plant responses to alkaline and saline stresses is essential for devising effective plant breeding and biotechnology strategies aimed at enhancing crop resilience and productivity in challenging environments.

Polyploidization represents a crucial evolutionary strategy that has allowed plants to cope with environmental stress and adapt to a wide range of habitats. As such, polyploid plants are of great interest to researchers and breeders for their potential to improve crop performance, resilience, and productivity in the face of climate change and other environmental challenges [8]. Polyploidization is often a result of whole-genome duplication (WGD), which can be induced naturally or artificially. It is considered the main driving force of plant evolution and the source of species diversity [9]. Polyploid plants have a rich genetic basis and can adapt to and withstand various environmental stress factors better than their diploid counterparts due to intricate molecular processes, including genetic redundancy, greater phenotypic plasticity, epigenetic regulation, and enhanced metabolic capacity [10,11]. Numerous studies have highlighted the role of polyploidization growth and productivity, which is adversely affected by saline–alkali conditions [12]. In our previous study, we clearly demonstrated that autotetraploid rice exhibited greater tolerance than diploid rice during the seeding stage when subjected to short-term salt stress (NaCl), alkali stress (NaOH), and saline–alkali stress (Na_2_CO_3_), respectively [13,14,15]. Under various saline–alkali stress conditions, polyploid and diploid rice manifested distinct stress response mechanisms. The increased tolerance of polyploid rice to saline–alkali stress was considered to be related to the changes in the polyploid genome or transcriptome [16]. The advent of genome-wide transcriptomic analysis has revolutionized the study of gene expression, enabling researchers to examine the entire transcriptome of an organism under specific conditions [11,17,18]. By leveraging the power of transcriptomic analysis, researchers can gain a more nuanced understanding of the molecular mechanisms that govern plant responses to saline–alkaline stress, which may ultimately lead to the development of stress-resistant crop varieties and improved agricultural practices in challenging environments.

In the presence of salt stress, transcriptome analysis revealed the participation of a large number of genes and quantitative trait loci (QTLs) in the biological regulatory process [19,20]. Transcription factors (TFs), as regulatory elements of gene transcription activation or inhibition, are significant regulators of plant responses to adverse conditions, including various types of abiotic stress, enabling plants to adapt and survive in challenging environments by modulating the expression of stress-responsive genes [21,22]. Many important TFs have site-specific conserved amino acids and other conserved domains, which contribute to their functionality and specificity. These conserved features allow TFs to be classified into distinct transcription factor families. An example of the role of TFs in stress response is the overexpression of *GmMYB3a* in transgenic soybeans, which was found to enhance the sensitivity of plants to salt and alkali stress [23]. Similarly, *ChbZIP1* in transgenic Arabidopsis mediated plant adaptation to alkali stress by facilitating the detoxification pathway of reactive oxygen species (ROS), thereby improving plant resistance to alkali stress [24]. *OsWRKY87* enhanced rice salt tolerance through synergistic interactions [25], while OsZFP213 worked in conjunction with OsMAPK3 to regulate salt tolerance in rice by increasing the capacity of ROS scavenging [26]. Plants overexpressing *OsMsr9* in Arabidopsis and rice exhibited a stronger tolerance to salt stress [27]. TFs such as MYB [28], bZIP [29], WRKY [30], and C2H2 [31] all function under abiotic stress to improve plant resistance. In this study, we conducted a transcriptome analysis to explore the differences in TF expression profiles between autotetraploid rice and its diploid donor under three distinct types of saline–alkali stress conditions. Our focus was on the expression of all transcription factor-related genes across the rice genome in order to analyze the possible molecular mechanisms underpinning the enhanced stress tolerance of tetraploid rice. This study contributes a valuable reference for the development of stress-resistant rice varieties, and also lays the groundwork for enhancing abiotic stress tolerance in polyploid plants. 

## 2. Materials and Methods

### 2.1. Plant Materials and Stress Treatments

*Oryza sativa* L. ssp. *Indica* ‘Yangdao 6′ cultivar 93–11, a diploid rice herein referred to as 9311-2x, and its corresponding autotetraploid line, herein referred to as 9311-4x, both of which have been described in previous articles [13], were employed. Seedlings at the 3-leaf stage were subjected to salt stress (NaCl), alkali stress (NaOH), and saline–alkali stress (Na_2_CO_3_) for 6 h (h). Leaf samples were then collected from these stress-treated seedlings, including 9311-2x (2X_NaCl, 2X_NaOH, and 2X_Na_2_CO_3_) and 9311-4x (4X_NaCl, 4X_NaOH, and 4X_ Na_2_CO_3_), as well as those not subjected to stress treatment, including 9311-2x (2X_Mock) and 9311-4x (4X_Mock), as previously described [13,14,15].

### 2.2. RNA Isolation and RNA-seq

Total RNA was extracted from the collected leaf tissues using the TRIzol reagent (Life Technologies Invitrogen, Carlsbad, CA, USA) according to the manufacturer’s protocol. The RNA concentration, purification, absorbance of nucleic acids, and integrity were assessed using the NanoDrop 2000 (Thermo Scientific, Waltham, MA, USA) and Agilent 2100 Bioanalyzer systems (Agilent, Waldbronn, Germany). Following genomic DNA elimination using RNase-free DNaseI, the RNA was reverse-transcribed with Super Script RNAse H reverse transcriptase, and cDNA library preparation and subsequent sequencing were conducted using the Illumina RNA-sequencing (RNA-seq) platform (Illumina, San Diego, CA, USA). The clean reads were acquired as described in previous studies [13,14,15]. To ensure that these reads were of sufficient quality for accuracy, we set up 24 runs (three repetitions for each operation) prior to data analysis. In each run, the total number of pair-end reads in the clean data was more than 21.9 million, the total alkali base in the clean data was more than 6.5 billion, the GC content was more than 55.36%, and the Q30 was more than 89.27% (Table S1). Gene expression levels were calculated using the FPKM (fragments per kilobase of transcript per million fragments mapped) method.

### 2.3. Data Analysis

The information on TFs was acquired from the Plant Transcription Factor Database (http://planttfdb.gao-lab.org/, accessed on 5 May 2022). The differentially expressed transcription factor (DETF) genes were functionally classified and enriched by GO (Gene Ontology) to analyze their biological processes, cellular components, and molecular function with a threshold of significance of adjusted *p*-value < 0.01. The correlation of biological duplication in the transcriptome sequencing data was evaluated using Pearson’s correlation coefficient (*r*^2^) and calculated using the R statistical package. Plots and gene expression heat maps were generated based on gene expression levels or log^2^ values using TBtools software, and the statistical significance threshold was set at *p* < 0.01. The differentially expressed genes (DEGs) were identified with an adjusted *p*-value < 0.01 and |log_2_ (fold change)| > 1.

### 2.4. qRT-PCR Validation

qRT-PCR was used to validate the RNA-seq data, the results of which are listed in Figure S1. Eight genes were selected randomly. The SYBR Green I PCR master mix kit (TaKaRa, Tokyo, Japan) was used in the qRT-PCR reactions. The experiment comprised three biological replicates. The primers used are listed in Table S2.

## 3. Results

### 3.1. Genome-Wide Expression of TFs-Related Genes Varied between 9311-2x and 9311-4x Rice under Saline–Alkali Stress

To explore the potential functional role of TFs in 9311-2x and 9311-4x rice plants in response to saline, alkaline, and saline–alkali stresses, we conducted a comparative transcriptomic analysis of the leaves sampled from these plants after each stress treatment for 6 h.

**Figure 1:**
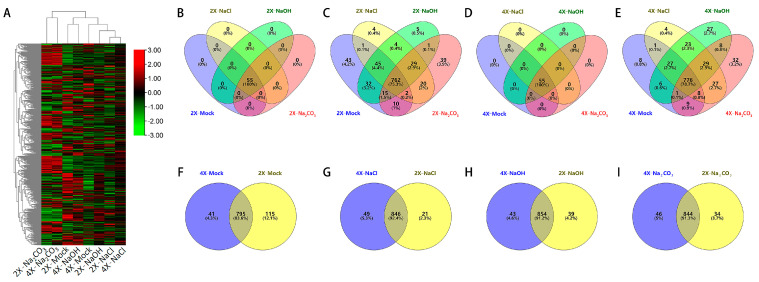
The data analysis of the 9311-2x and 9311-4x leaves under Mock, NaCl stress, NaOH stress, and Na_2_CO_3_ stress, respectively. (**A**) Heatmap of the related genes expression patterns from the 56 TF families. (**B**) Venn diagrams of the number of TF families expressed in the 9311-2x leaves under both mock and stress treatments. (**C**) Venn diagrams of the TF genes expressed in the 9311-2x leaves under four types of treatment. (**D**) Venn diagrams of the number of TF families expressed in the 9311-4x leaves under both mock and stress treatments. (**E**) Venn diagrams of the TF genes expressed in the 9311-4x leaves under both mock and stress treatments. Venn diagrams of the TF genes expressed in the 9311-4x and 9311-2x leaves in mock (**F**), NaCl stress (**G**), NaOH stress (**H**), and Na_2_CO_3_ stress (**I**).

A total of 56 transcription factor families were identified in the rice leaf transcriptome based on the standard of count > 30 (Figure 1A). Except for LFY (LEAFY), significant variations in the expression of these TFs were discernible between 9311-2x and 9311-4x upon three types of stresses, with the Na_2_CO_3_ stress being the most prominent (Figure 1B,D, Table S3). Among the represented transcription factor families, bHLH, bZIP, MYB, WRKY, and C2H2 were the top five TF families highly expressed, with the bHLH family being the most abundant. A total of 762 TF-related genes were co-expressed following NaCl stress, NaOH stress, and Na_2_CO_3_ stress in 9311-2x leaf tissues (Figure 1C), with the number of TF genes expressed in the three stress treatment groups being lower than that in the control group (Table S3). In contrast, 776 TF genes were co-expressed in 9311-4x leaf samples under both mock and three types of stress treatments (Figure 1E). There were 795 TF genes that were expressed in both 9311-2x and 9311-4x, 41 TF genes that were specific to 9311-4x, and 115 TF genes that were specific to 9311-2x (Figure 1F). Consequently, there were 846 common, 49 9311-4x-specific, and 21 9311-2x genes under NaCl stress (Figure 1G); 854 common, 43 9311-4x, and 39 9311-2x genes under NaOH stress (Figure 1H); and 844 common, 46 9311-4x, and 34 9311-2x genes under Na_2_CO_3_ stress (Figure 1I).

The number of TF genes that were co-expressed in both the 9311-2x and 9311-4x leaves was higher under the three types of stress conditions than that in the mock condition (Figure 1F–I, Table S3). Interestingly, the number of TF-related genes expressed in the 9311-4x leaves was higher than that of the 9311-2x leaves under three types of stress (Figure 1F–I). It is evident that specific variations in the expression of TF genes exist between 9311-2x and 9311-4x, and that the amplitude of these differences varies according to stress conditions.

### 3.2. Analysis of Differentially Expressed 56 Transcription Factor Families’ Genes in Diploid and Autotetraploid Rice Leaves

**Figure 2:**
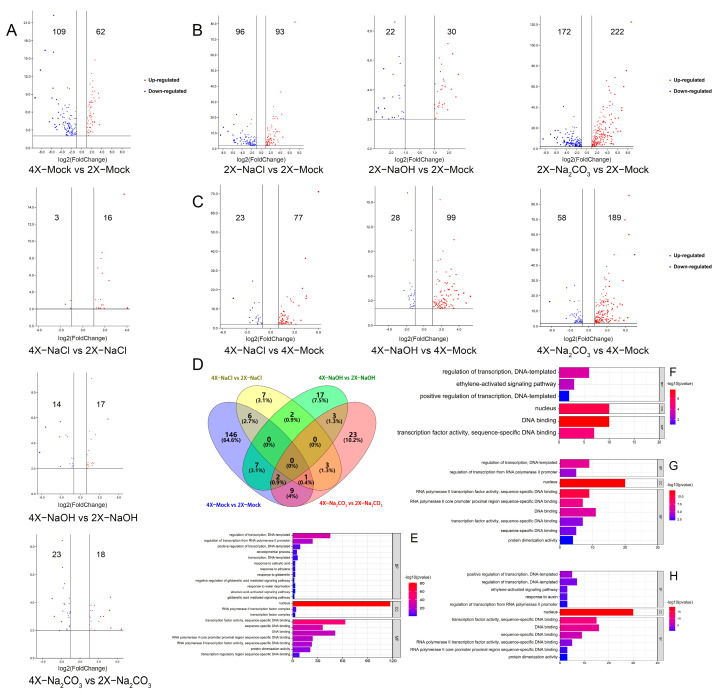
(**A**) Volcanic map of differentially expressed transcription factor (DETF) genes comparing 9311-2x and 9311-4x leaves in response to mock, NaCl, NaOH, and Na_2_CO_3_ treatments. (**B**) Volcanic map of DETF genes in 9311-2x in response to mock, NaCl, NaOH, and Na_2_CO_3_ treatments. (**C**) Volcanic map of DETF genes in 9311-4x in response to treatments and control under NaCl, NaOH, and Na_2_CO_3_. (**D**) Venn diagram of DETF genes comparing 9311-2x and 9311-4x in response to mock, NaCl, NaOH, Na_2_CO_3_ treatments. (**E**–**H**) GO enrichment map of DETF genes comparing 9311-2x and 9311-4x in response to mock control, NaCl, NaOH, and Na_2_CO_3_.

The DEGs encoding TFs in the 9311-4x and 9311-2x leaves in response to NaCl, NaOH, and Na_2_CO_3_ stress treatments were profiled and compared. Under the mock condition, a total of 171 differentially expressed transcription factor (DETF) genes, including 109 downregulated and 62 upregulated, were identified across 34 TF families between the leaf samples derived from two ploidy levels (4X_Mock vs. 2X_Mock) (Figure 2A). Upon NaCl treatment, 19 DETF genes, including 16 upregulated and four downregulated, were identified across twelve TF gene families between the leaves of two ploidy levels (4X_NaCl vs. 2X_NaCl) (Figure 2A). Upon NaOH treatment, a total of 31 DETF genes, including 17 upregulated and 14 downregulated, were identified across 16 TF gene families between the leaves of ploidy levels (4X_NaOH vs. 2X_NaOH) (Figure 2A). Upon Na_2_CO_3_ treatment, 41 DETF genes, including 18 upregulated and 23 downregulated, were identified across 16 TF families between the leaves of two ploidy levels (4X_Na_2_CO_3_ vs. 2X_Na_2_CO_3_) (Figure 2A). In the 9311-2x leaves, there were 189 (93 upregulated and 96 downregulated), 52 (30 upregulated and 22 downregulated), and 394 (222 upregulated and 172 downregulated) differentially expressed genes upon NaCl, NaOH, and Na_2_CO_3_ stress treatments, respectively (Figure 2B). In contrast, there were 100 (77 upregulated and 23 downregulated, 127 (99 upregulated and 28 downregulated), and 247 (189 upregulated and 58 downregulated) DETF genes upon NaCl, NaOH, and Na_2_CO_3_ stress treatments, respectively (Figure 2C).

The NA co-expression of DETF genes is intriguing in 4X_Mock vs. 2X_Mock, 4X_NaCl vs. 2X_NaCl, 4X_NaOH vs. 2X_NaOH, and 4X_Na_2_CO_3_ vs. 2X_Na_2_CO_3_, as revealed by the Venn map analysis (Figure 2D). The GO enrichment analysis of 4X_Mock vs. 2X_Mock showed that three TF genes were enriched in the “response to water deprivation” pathway, and numerous TF genes that were enriched in the gibberellin, salicylic acid, abscisic acid, and ethylene response pathways (Figure 2E, Table S4). Likewise, 4X_NaCl vs. 2X_NaCl showed that three TF genes were enriched in the ethylene activation signal pathway (Figure 2F, Table S4). In contrast, no TF genes were increased in other pathways, apart from the pathway related to transcription regulation in the biological process involved in 4X_NaOH vs. 2X_NaOH (Figure 2G, Table S4). Three TF genes were enriched in each of the auxin response pathways and the ethylene activation signal pathway (Figure 2H, Table S4).

### 3.3. Comparative Analyses of the DETF Genes in Rice Induced by Salt, Alkali, and Saline–Alkali Stress Treatments

**Figure 3:**
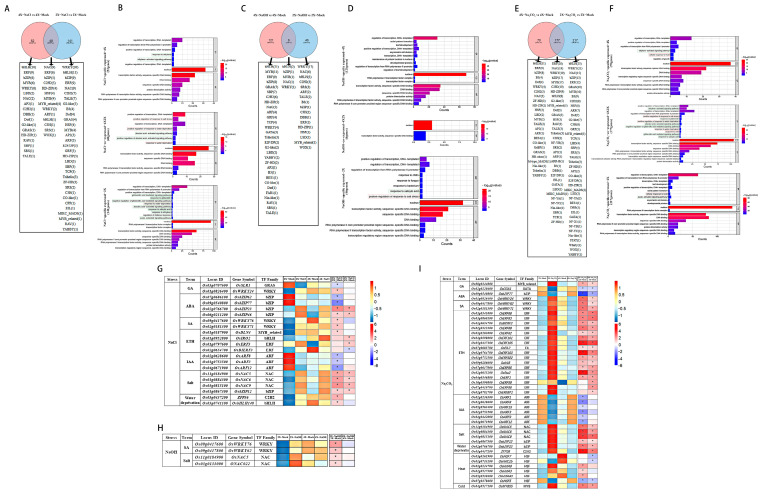
Venn diagrams of TF families between treatment and control under (**A**) NaCl stress, (**C**) NaOH stress, and (**E**) Na_2_CO_3_ stress. The relative TF families and the number of transcription factor family members are listed in the square frame below. The GO enrichment of DETF genes under (**B**) NaCl stress, (**D**) NaOH stress, and (**F**) Na_2_CO_3_ stress. From top to bottom, 9311-2x-specific TFs, common DETF genes of 9311-2x and 9311-4x, and 9311-4x-specific TFs are represented. Heatmaps of expression levels and log2 values were made using TFs and were enriched into hormone-related and abiotic stress-related pathways in 9311-2x and 9311-4x under (**G**) NaCl stress, (**H**) NaOH stress, and (**I**) Na_2_CO_3_ stress. * *p* < 0.05.

The DETF genes induced in rice by salt, alkali, and saline–alkali stress treatments showed were comparatively investigated. In response to salt stress, 48 DETF genes from 14 TF families were co-expressed in 4X_NaCl vs. 4X_Mock and 2X_NaCl vs. 2X_ Mock, 52 DETF genes from 18 TF families were specifically expressed in 4X_NaCl vs. 4X_Mock, and 141 DETF genes from 30 TF families were specifically expressed in 2X_NaCl vs. 2X_ Mock (Figure 3A). The GO analysis showed that the positive regulation of the response to salt stress, the positive regulation of the response to water deprivation, and the abscisic acid-activated signaling pathway were enriched and commonly expressed in 4X_NaCl vs. 4X_Mock and 2X_NaCl vs. 2X_ Mock (Figure 3B). The response to gibberellin, response to salicylic acid, and response to ethylene pathway were enriched in specifically expressed in 4X_NaCl vs. 4X_Mock (Figure 3B). The response to ethylene and the ethylene-activated signaling pathway were enriched and specifically expressed in 2X_NaCl vs. 2X_Mock (Figure 3B, Table S4). In response to alkali stress, there were 6 DETF genes from 5 TF families co-expressed compared to 4X_NaOH vs. 4X_Mock and 2X_NaOH vs. 2X_ Mock, 121 DETF genes from 28 TF families specifically expressed in 4X_NaOH vs. 4X_Mock, and 46 DETF genes from 17 TF families specifically expressed in 2X_NaOH vs. 2X_ Mock, respectively (Figure 3C). The TF families are listed and arrowed in the Venn diagram below, and the DETF genes are labelled following the TF families (Figure 3C). Accordingly, the response to salicylic acid and the positive regulation of the response to salt stress pathways were enriched and specifically expressed in 2X_NaOH vs. 2X_Mock, as determined via GO enrichment analysis (Figure 3D, Table S4). In response to saline–alkali stress, there were 177 DETF genes from 35 TF families co-expressed compared to 4X_ Na_2_CO_3_ vs. 4X_Mock and 2X_ Na_2_CO_3_ vs. 2X_ Mock, 70 DETF genes from 25 TF families specifically expressed in 4X_ Na_2_CO_3_ vs. 4X_Mock, and 217 DETF genes from 40 TF families specifically expressed in 2X_ Na_2_CO_3_ vs. 2X_ Mock, respectively (Figure 3E). The TF families are listed and arrowed in the Venn diagram below, and the DETF genes are labelled following the TF families (Figure 3E). The ethylene-activated signaling, the positive regulation of the response to salt stress, and the response to gibberellin pathways were enriched and commonly expressed in 4X_ Na_2_CO_3_ vs. 4X_Mock and 2X_ Na_2_CO_3_ vs. 2X_ Mock (Figure 3F, Table S4). The ethylene-activated signaling pathway and regulation of growth pathway were enriched and specifically expressed in 4X_Na_2_CO_3_ vs. 4X_Mock (Figure 3F, Table S4) The leaf development and auxin-activated signaling pathways were enriched and specifically expressed in 2X_ Na_2_CO_3_ vs. 2X_Mock, as determined via GO enrichment analysis (Figure 3F, Table S4).

To further study the functions of these DETF genes, we compiled the related genes involved in the phytohormone responses and abiotic stress responses under salt stress, alkali stress and saline–alkali stress treatments (Figure 3G–I, Table S5). The upregulated TF genes in response to these abiotic stresses encompass the WRKY family (related to SA), ERF family (related to ETH), and NAC family (related to salt stress). However, there were distinct responses between diploid and autotetraploid rice under these stresses; for example, the ARF family (related to IAA) was downregulated only in diploid rice (Figure 3G–I, Table S5).

**Figure 4:**
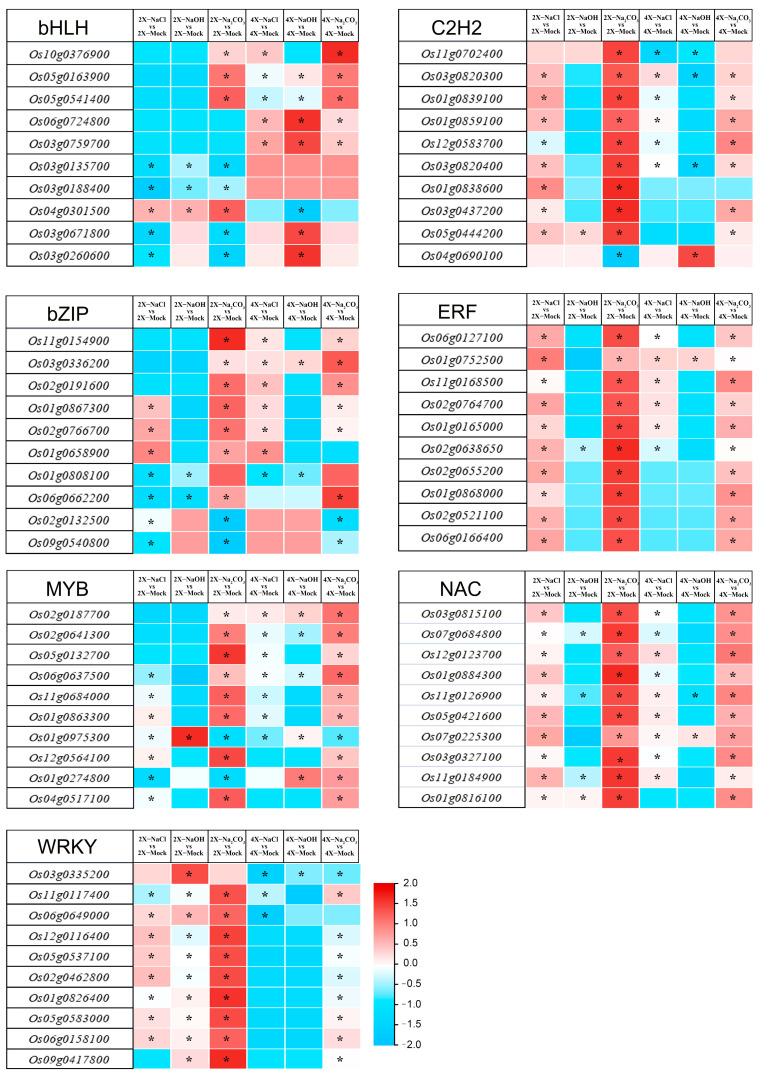
Heatmap of the DETF genes of bHLH, bZIP, MYB, WRKY, C2H2, ERF, and NAC (Top 10) with 9311-2x and 9311-4x treatments with control groups under NaCl stress, NaOH stress, and Na_2_CO_3_ stress. * *p* < 0.05.

Based on the results of the DETF genes between 9311-2x and 9311-4x following the three types of saline–alkali stress, the TF families bHLH, bZIP, MYB, WRKY, and C2H2, exhibited the greatest change induced by stress. Moreover, some of the TF families, particularly in the ERF- and NAC-related genes, expressed variation significantly following stress. We analyzed and listed the related DETF genes with fold change (log value, base 2) in Figure 4 (Top 10) and Supplementary Table S5. After the three types of saline–alkali stress, the DETF genes in the 9311-2x and 9311-4x leaves mainly had biological stress functions in the abiotic stress response and phytohormone signaling pathways, and the variations in the expression of the related genes were different among salt stress, alkali stress and saline–alkali stress.

### 3.4. Identification of the Common Expressed DETF Genes in the 9311-2x and 9311-4x Leaves in Response to Salt, Alkali, and Saline–Alkali Stress

**Figure 5:**
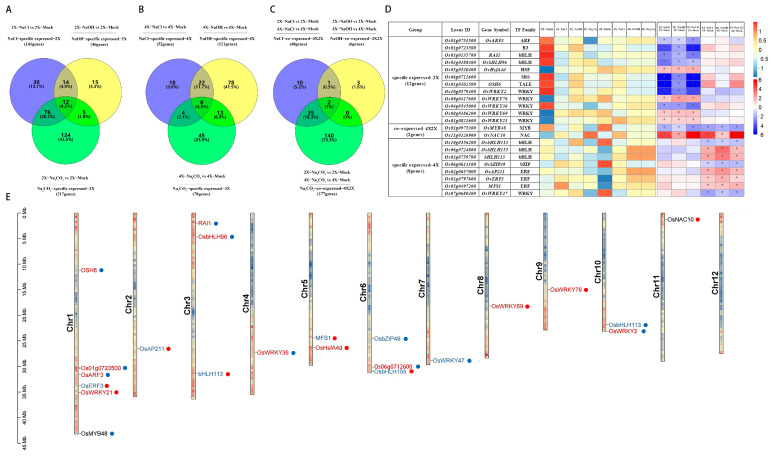
Among the three stresses, Venn diagrams of (**A**) DETF genes of 9311-2x stress groups and control group, (**B**) DETF genes of 9311-4x stress groups and control group, (**C**) DETF genes co-expressed in 9311-2x and 9311-4x, (**D**) Heatmap of co-expressed and specifically expressed DETF genes in 9311-2x and 9311-4x, respectively. * *p* < 0.05. (**E**) The 12 chromosomes of rice are represented with color bars, and different colors in the same color bar represent the enrichment of genes at the location of the chromosome. The position of TFs corresponds to the scale on the left. The 2 black gene names represent the co-expression of DETF genes in 9311-2x and 9311-4x under the three stresses, while the 12 red gene names represent the unique expression of DETF genes in 9311-4x plants under the three stresses. The eight blue gene names represent the unique expression of DETF genes in 9311-2x under the three stresses. Red dots represent gene upregulation, and blue dots represent gene downregulation.

The TF genes that are specifically expressed in diploid rice were identified under NaCl stress (141 genes, Figure 3A), under NaOH stress (46 genes, Figure 3C), and under Na_2_CO_3_ stress (217 genes, Figure 3E). The Venn diagram analyzed revealed that there were 12 DETF genes expressed in 9311-2x in response to salt, alkali, and saline–alkali stress when compared with the DETF genes in 2X_ NaCl vs. 2X_Mock, 2X_ NaOH vs. 2X_ Mock, and 2X_ Na_2_CO_3_ vs. 2X_ Mock (Figure 5A). The 12 commonly expressed DETF genes belonged to 7 TF families (ARF, B3, bHLH, HSF, SRS, TALE, and WRKY), whose expression pattern is listed in Figure 5D (Table S5); in addition, the heatmap results showed that most of the genes were downregulated by saline–alkali stress in diploid rice. Meanwhile, there were only 39 uniquely expressed DETF genes induced by NaCl stress in 9311-2x, only 15 uniquely expressed DETF genes induced by NaOH stress in 9311-2x, and only 124 uniquely expressed DETF genes induced by Na_2_CO_3_ stress in 9311-2x. Additionally, the TF genes that are specifically expressed in autotetraploid rice were identified under NaCl stress (52 genes, Figure 3A), under NaOH stress (121 genes, Figure 3C), and under Na_2_CO_3_ stress (70 genes, Figure 3E). The Venn diagram analyzed revealed that there were eight DETF genes expressed in 9311-4x in response to salt, alkali, and saline–alkali stress when compared with the DETF genes in 4X_ NaCl vs. 4X_Mock, 4X_ NaOH vs. 4X_ Mock, and 4X_ Na_2_CO_3_ vs. 4X_ Mock (Figure 5B). The eight commonly expressed DETF genes belonged to four TF families (bHLH, bZIP, ERF, and WRKY), whose expression pattern is listed in Figure 5D (Table S5); in addition, the heatmap results showed that most of the genes were upregulated by saline–alkali stress in autotetraploid rice. Meanwhile, only 18 uniquely expressed DETF genes were induced by NaCl stress in 9311-2x, only 38 uniquely expressed DETF genes were induced by NaOH stress in 9311-2x, and only 45 uniquely expressed DETF genes were induced by Na_2_CO_3_ stress in 9311-2x (Figure 5B). Furthermore, the TF genes that are co-expressed in diploid and autotetraploid rice were identified under NaCl stress (48 genes, Figure 3A), under NaOH stress (six genes, Figure 3C), and under Na_2_CO_3_ stress (177 genes, Figure 3E). The Venn diagram analyzed revealed that there were two DETF genes commonly expressed in 9311-2x and 9311-4x in response to salt, alkali, and saline–alkali stress when compared with the DETF genes in 2X_ NaCl vs. 2X_Mock VS 4X_ NaCl vs. 4X_Mock, 2X_ NaOH vs. 2X_Mock VS 4X_ NaOH vs. 4X_Mock, and 2X_ Na_2_CO_3_ vs. 2X_Mock VS 4X_ Na_2_CO_3_ vs. 4X_Mock (Figure 5C). The two commonly expressed DETF genes belonged to two TF families (MYB and NAC), whose expression pattern is listed in Figure 5D (Table S5). Meanwhile, only 10 uniquely expressed DETF genes were induced by NaCl stress in 9311-2x and 9311-4x, only 3 uniquely expressed DETF genes were induced by NaOH stress in 9311-2x and 9311-4x, and only 140 uniquely expressed DETF genes were induced by Na_2_CO_3_ stress in 9311-2x and 9311-4x (Figure 5C). The distribution of the 22 commonly expressed DETF genes was mapped to 12 chromosomes across the rice genome (Figure 5E).

## 4. Discussion

Research on the abiotic stress resistance of rice primarily focuses on drought, salinity, and cold stress. Although numerous studies have explored the molecular mechanisms underlying saline–alkali tolerance in rice, investigations into the distinctions between salt tolerance and alkali tolerance remain limited. In this study, we performed a comparative analysis of TF genes expressed in the 9311-2x and 9311-4x rice leaves under different growth environments. We identified the critical TF genes that are co-expressed in both, as well as those that are uniquely expressed under NaCl, NaOH, and Na_2_CO_3_ stress. By comprehensively comparing and analyzing the TF genes of the 9311-2x and 9311-4x leaves under three different saline–alkali pressures, this study offers a valuable reference for understanding the molecular mechanisms underlying salt tolerance in polyploid rice.

TFs regulate chromatin and transcription by recognizing specific DNA sequences, thus forming intricate systems that direct genome expression. TFs can be divided into various families, such as MYB, bZIP, WRKY, bHLH (basic helix–loop–helix), C2H2, and others [28,29,30,31,32]. In this study, 55 TF families were expressed in the 9311-2x and 9311-4x leaves under NaCl stress, NaOH stress, and Na_2_CO_3_ stress, with no members of the LFY family being expressed. The top five TF families in terms of representation were bHLH, bZIP, MYB, WRKY, and C2H2. The response of transcription factors differed in the diploid and autotetraploid rice leaves under saline–alkali stress conditions. Under NaCl stress, 241 members from 34 TF families were differently expressed in the 9311-2x and 9311-4x leaves. Under NaOH stress, 173 members from 36 TF families were differentially expressed in the 9311-2x and 9311-4x leaves. Under Na_2_CO_3_ stress, the 464 TF genes differentially expressed in the 9311-2x and 9311-4x leaves belonged to 47 transcription factor families. The CPP family was differentially expressed only under NaCl stress, the FAR1 family only under NaOH stress, and nine TF families, such as M-type_MADS, were only differentially expressed under Na_2_CO_3_ stress. These results demonstrate that different TF families have distinct regulatory mechanisms when faced with various stresses. The network regulation mechanism under Na_2_CO_3_ stress may be more complex than that under NaCl and NaOH stress.

There are specific transcription factors responsive to saline–alkali stress in the diploid and autotetraploid rice leaves, respectively. For example, there were 52 specific DETF genes under NaCl stress, 121 specific DETF genes under NaOH stress, and 70 specific DETF genes under Na_2_CO_3_ stress in autotetraploid rice. Furthermore, the Venn analysis showed that only eight genes were expressed commonly among NaCl stress, NaOH stress, and Na_2_CO_3_ stress in autotetraploid rice, including the genes of *OsbHLH155(Os06g0724800)*, *bHLH113(Os03g0759700)*, *OsAP211(Os02g0657000)*, *OsERF3(Os01g0797600)*, and *MFS1(Os05g0497200),* which were upregulated under saline–alkali stress, and the genes of *OsbHLH113(Os10g0556200)*, *OsbZIP49(Os06g0614100)*, and *OsWRKY47(Os07g0680400)*, which were downregulated under saline–alkali stress. There are many transcription factors that show a differing response to saline–alkali stress in both the diploid and autotetraploid rice leaves. Interestingly, the gene of *Os05g0579600* (MYB family) was downregulated by two-fold in 9311-2x and downregulated by six-fold in 9311-4x under NaCl stress. The gene of *Os04g0664400* (ARF family) was downregulated by 12-fold in 9311-2x and upregulated by 9-fold in 9311-4x under NaCl stress. The gene of *Os03g0741100* (bHLH family) was upregulated by two-fold in 9311-2x and downregulated by six-fold in 9311-4x under NaCl stress. Thus, the expression patterns of the transcription factors in the diploid and autotetraploid rice leaves under saline–alkali stress were different; the expression of each TF gene requires specific analysis in diploids and autotetraploid rice.

We conducted a GO enrichment of DETF genes under three different stress factors and observed that the biological functions of TFs in the 9311-2x and 9311-4x leaves were not only related to transcription, but also enriched in hormone signaling pathways and abiotic stress response pathways such as salt, high temperature, cold, and water deficiency. Among them, *OsNAC5* was found to be enriched in the “positive regulation of response to salt stress” pathway under all three stresses except the 4X_NaOH treatment group. Additionally, three TF genes were identified as being enriched in the same pathway under NaCl and Na_2_CO_3_ treatment but not in NaOH treatment. Another TF, *OsWRKY76,* was enriched in the “response to salicylic acid” pathway under NaCl and NaOH treatment. The hormonal signaling pathways and abiotic stress response pathways in the 9311-2x and 9311-4x leaves were highly represented under all three stress factors. Furthermore, we also identified DETF genes that were co-expressed in the 9311-2x and 9311-4x leaves under all three stress factors, as well as DETF genes that were specific to each of the 9311-2x and 9311-4x leaves. These genetic resources could serve as valuable references for understanding the biological functions and improving the genetic tolerance of rice to saline–alkali stress.

Several TF families, such as MYB [22,33], bZIP [34] and NAC [35], have been found to improve plant stress resistance, particularly in their ability to mitigate salt and alkali stress. The overexpression of *GmMYB3a* in transgenic soybean has been shown to increase plant sensitivity to salt and alkali stress [23]. *ChbZIP1* overexpression in transgenic Arabidopsis can mediate plant adaptation to alkali stress through the detoxification pathway of ROS, thus enhancing plant resistance to alkali stress [24]. Similarly, the overexpression of *OsMYB48-1* can significantly improve rice’s resistance to drought and salt stress [36], and *OsNAC10* enhances rice’s tolerance to drought, high salt, and low temperature [37]. In our study, many TF families, such as bZIP, MYB, WRKY, C2H2, and NAC, were differentially expressed under three different salt and alkali stress treatments in 9311-2x and 9311-4x. In particular, *OsMYB48* was consistently downregulated and *OsNAC10* was upregulated more than two-fold in all six comparison groups. Notably, both 9311-2x and 9311-4x were upregulated by more than 4-fold under NaCl stress, and more than 64-fold under Na_2_CO_3_ stress, suggesting that there are distinct response mechanisms in the diploid and tetraploid rice leaves under these stress conditions. Interestingly, our findings differ from previous studies in terms of the differential expression of *OsMYB48*, which may be due to different concentrations of NaCl stress treatment. Meanwhile, our study confirms the positive regulatory role of *OsNAC10* in response to salt and alkali stress, especially under NaCl and Na_2_CO_3_ stress. We also investigated the DETF genes under saline–alkali stress in the root of diploid and autotetraploid rice in a previous report [38]; there were many DETF genes related to the phytohormone response, however, due to the tissue specificity of gene expression, we found that there were significant differences between the roots and leaves of rice under saline–alkali stress. It was therefore necessary to study the DETF genes that respond to saline–alkali stress in the leaves of rice. Although many genes related to salt and alkali stress treatment have been studied, research related to alkali resistance and saline—alkali tolerance remains limited. Thus, the DETF genes identified in this study can serve as valuable genetic resources and references for further studies on salt and alkali tolerance in both diploid and autotetraploid rice.

## 5. Conclusions

Our study found significant variation in the expression of TF genes in the 9311-2x and 9311-4x leaves under different growth environments, with abundant DETF genes under three different salt and alkali stress treatments. DETF genes were enriched not only in transcription-related pathways, but also in plant hormone signal transduction and abiotic stress-related pathways, particularly in response to salt stress. Our findings provide strong evidence that autotetraploid rice is more resistant to salt and alkali stress than diploid rice. These findings offer valuable insights for further investigation into the differences in the regulatory mechanisms of stress resistance between diploid and polyploid plants. Further, it can inform crop breeding efforts that are aimed at developing more stress-tolerant rice varieties. Finally, this study may contribute to a broader understanding of polyploid evolution and its implications for plant adaptation to changing environments. Polyploidization is a common phenomenon in plants, and investigating the differences in the stress response between diploid and autotetraploid rice may provide insights into the adaptative value of polyploidization in plants.

## Data Availability

The datasets generated and analyzed in this study are available at PRJNA856424 (https://www.ncbi.nlm.nih.gov/sra/PRJNA856424, accessed on 5 May 2022), PRJNA812638 (https://www.ncbi.nlm.nih.gov/sra/PRJNA812638, accessed on 8 July 2022) and PRJNA873237 (https://www.ncbi.nlm.nih.gov/bioproject/?term=PRJNA873237, accessed on 26 August 2022).

## Supplementary Materials

The following supporting information can be downloaded at: https://www.mdpi.com/article/10.3390/genes14061151/s1, Table S1: RNA-seq data of the diploid and tetraploid rice leaves under NaCl stress, NaOH stress and Na_2_CO_3_ stress; Table S2: List of primers of qPCR; Table S3: List of transcription factor families in diploid and tetraploid rice identified by transcriptome analysis among NaCl stress, NaOH stress and Na_2_CO_3_ stress. Table S4: The data for GO enrichment analysis. Table S5: The data for heatmap analysis. Figure S1: The results of qPCR.
