# Supplementary figures and images for: Evaluating the Differential Response of Transcription Factors in Diploid versus Autotetraploid Rice Leaves Subjected to Diverse Saline–Alkali Stresses
Source: Genes (Basel). 2023 May 25;14(6):1151. doi: 10.3390/genes14061151 (PMC10298515; doi:10.3390/genes14061151)

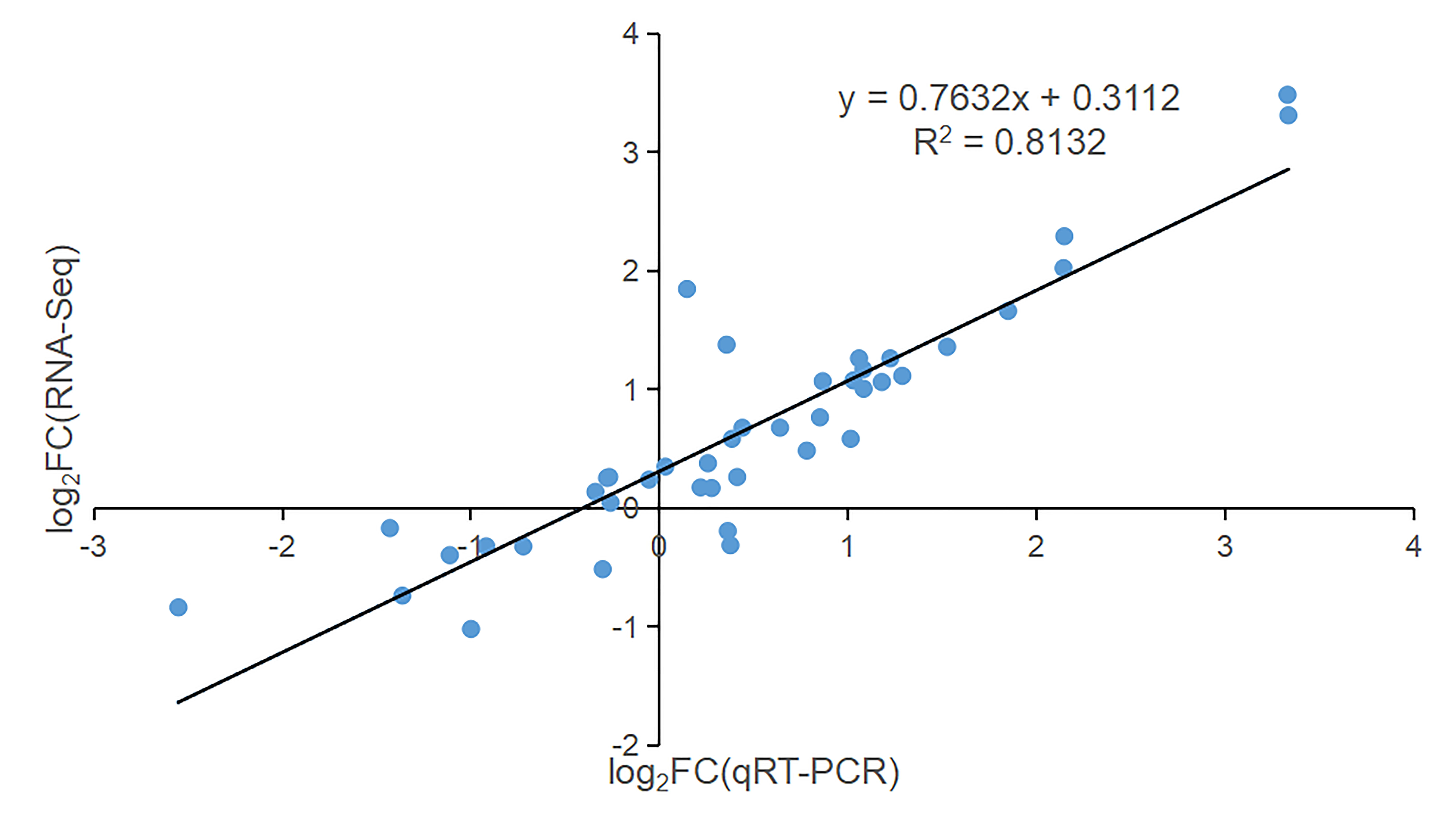

Supplement: Supplementary file 1 [file genes-14-01151-s001.zip › Supplementary Figure S1.jpg]
